# Detection of an Abundant Plant-Based Small RNA in Healthy Consumers

**DOI:** 10.1371/journal.pone.0137516

**Published:** 2015-09-03

**Authors:** Jian Yang, Lisa M. Farmer, Abia A. A. Agyekum, Ismail Elbaz-Younes, Kendal D. Hirschi

**Affiliations:** 1 USDA/ARS Children's Nutrition Research Center, Baylor College of Medicine, Houston, Texas, United States of America; 2 Vegetable and Fruit Improvement Center, Texas A&M University, College Station, Texas, United States of America; Huazhong university of Science and Technology, CHINA

## Abstract

The mechanisms of delivery of plant small RNAs to consumers must be investigated in order to harness this technology to positively impact biotechnology. Two groups have used honeysuckle (*Lonicera japonica*) feeding regimes to detect a plant-based small RNA, termed MIR2911, in sera. Meanwhile, numerous groups have failed to detect dietary plant-based small RNAs in consumers. Here we catalog levels of MIR2911 in different herbs, and suggest that in particular herb MIR2911 levels are elevated. Feeding these different herb-based diets to mice, we found MIR2911 levels in the sera and urine were associated with dietary intake levels. Abundance was not the sole determinate of apparent RNA bioavailability, as gavage-feeding large-doses of synthetic MIR2911 permitted only small transient increases in serum levels. Dietary MIR2911 were not modified in circulation by association with the host’s RNA-induced silencing complex, as the RNA did not co-immunoprecipitate with AGO2. The stability of dietary MIR2911 in circulation differed from synthesized small RNAs, as tail vein administration of various synthetic plant-based small RNAs resulted in rapid clearance. However, synthetic MIR2911 appeared to be more stable than the other plant miRNAs tested. Notably, this uptake of dietary MIR2911 was not related to perturbations in the host’s microbiome or gut permeability. We suggest dietary uptake of MIR2911 is commonplace in healthy consumers, and reproducible detection of plant-based small RNAs in consumers depends on dietary abundance, RNA stability and digestion from within the food-matrix.

## Introduction

Nucleic acids from the diet are frequently used by molecular biologists to exert a strong influence on gene regulation in several organisms, most notably in worms (*C*. *elegans*), and the hypothesis that similar regulation could occur in our bodies has generated intense interest [1]. Both plants and animals contain hundreds of different small RNAs, including microRNAs (miRNAs) that are 19–24 nucleotides long [2]. In plants, miRNAs recognize their targets with essentially perfect complementarity and effect RNA cleavage and degradation [3], while in animals, the endogenous miRNAs inhibit translation and alter transcript stability by binding to target transcripts with largely imperfect complementarity [2]. A single type of miRNA can shift the transcript profile of a cell, consistent with a pivotal role for miRNAs in establishing and maintaining tissue identity [4]. In addition to functioning locally, a model has been suggested whereby miRNAs are encapsulated by extracellular vesicles (EVs), which are shed from almost all animal cell types and circulate to distant organs, with the potential to interact with specific target cells [5]. Argonaute2 (AGO2), the key effector protein of miRNA-mediated silencing, is often bound to the circulating miRNAs for enhanced serum stability [6]. Many endogenous small RNA species have been detected in circulation, and stable endogenous miRNAs in mammalian blood are emerging as a novel class of biomarkers for various diseases [7].

Small RNAs are abundant in numerous plant foods, and some display perfect complementarity to human genes [8]. A report challenging multiple paradigms, suggested that ingested plant-based miRNAs are transferred to blood, accumulate in tissues, and regulate endogenous gene expression in animals [9]. Subsequently, varying levels of success have been reported regarding detection of exogenous dietary miRNAs in mammalian consumers [10–16]. To date, plant-based dietary small RNAs have been difficult to detect in the sera of consumers fed a single serving of the food [15, 17].

In the midst of these findings, studies have measured circulating levels of a plant 26S ribosomal RNA-derived small RNA, previously referred to as miRNA MIR2911. A diet supplemented with Honeysuckle (*Lonicera japonica*), which naturally contains high levels of MIR2911, promotes the passage of MIR2911 through the lining of the mouse gastrointestinal (GI) tract, where it is then transferred to the bloodstream and subsequently, the lungs [18]. In honeysuckle-fed animals, MIR2911 reached high levels in circulation within three days of consumption and dissipated 48 hours after the honeysuckle was removed from the diet [19]. Given the abundance of MIR2911 in honeysuckle and its apparent stability, it represents a model small RNA to measure uptake parameters [18, 20]. In our previous study, a model has been proposed where the dietary consumption of honeysuckle promotes uptake of MIR2911 by affecting the permeability of the GI tract or influencing the microbiome within the GI tract [19]; however, this has not been experimentally tested. Alternatively, the uptake of MIR2911 may be a more commonplace phenomenon that can occur when eating a variety of plant-based foods. Here we have characterized MIR2911 levels in various foods and tested diets for their capacity to promote serum and urine detection of MIR2911. We assayed whether this dietary small RNA in peripheral blood could co-immunoprecipitate (co-IP) with a mouse anti-AGO2 antibody. We then examined dosage requirements and intestinal parameters that could impact MIR2911 detection. Establishing dietary factors capable of promoting nucleic acid absorption and retention is an invaluable foundation for future studies regarding functionality and gene-targeted oral therapeutics.

## Materials and Methods

### Animal studies

The IACUC of Baylor College of Medicine approved the mouse feeding studies and all other experimental procedures. All mice were obtained from the Center for Comparative Medicine at Baylor College of Medicine. Male ICR mice at 8- to 10-weeks-old were used in all feeding studies, which were replicated at least three times; the results shown are representative of the biological replicates. Herb and flower diets for mice were prepared from finely ground plant tissues obtained from various local Chinese herbal medicine and tonic stores. The plant-chow diets were prepared by mixing finely ground chow, plant material, and water at 2:1:2 weight ratios. Amoxicillin (50 mg/kg/day) and Trimethoprim Sulfa (TMS) (160 mg/kg/ml) were administered in the drinking water based on the assumption the mice were each drinking 5.0 ml of water per day [21]. Tail vein injections were done according to standard protocols [22]; 50 fmol of each RNA was resuspended in 100 μl of phosphate buffered saline (PBS) and injected into the lateral tail vein. Synthetic miRNAs were obtained from Integrated DNA Technologies. Antibiotics were obtained from Sigma.

### Serum and urine collection and RNA extraction

Blood was collected via retro-orbital bleeding of mice and was allowed to coagulate at room temperature for 1 h prior to sera isolation. Sera were separated by centrifugation at 800 x *g* for 10 min at room temperature followed by centrifugation at 10,000 x *g* at 4°C for 10 min to remove all blood cells and debris. Clean urine samples free of feces were collected by holding mice over parafilm and encouraging micturition [23]. Total RNA was extracted from 100 μL of sera or 80 μL of urine using miRNeasy Mini Kit from Qiagen following manufacturer’s recommendations. For urine samples, 1 pmol of synthetic MIR161 was spiked in as an exogenous RNA control.

### Analysis of miRNA levels by qRT-PCR

Taqman microRNA Assays for let-7dgi [24], miR-16, MIR161, MIR2911, MIR156a, MIR168a and artificial miRNA C7 were obtained from Life Technologies. Total RNA equivalent to 10 μL of sera or 8 μL of urine were used in each reverse transcription (RT) reaction. Of the 10 μL RT product, 0.5 μL was used for each triplicated quantitative polymerase chain reaction (PCR). To quantify miRNA levels in herbs and flowers, 10 mg of dried plant material were ground to fine powder in liquid nitrogen and then subjected to RNA isolation using the miRNEASY kit (Qiagen); 1 pmol of synthetic MIR161 was spiked into the plant Qiazol lysate as an exogenous RNA control. qRT-PCR was performed using a Biorad CFX96 Real-Time PCR Detection System, and data were analyzed using Biorad CFX software. Delta-Delta-Ct method was used to calculate relative levels of miRNAs. Absolute concentrations of miRNAs were calculated based on standard curves obtained from serial dilutions of synthetic miRNAs. To verify the fidelity of Taqman microRNA assay kit for MIR2911, the qPCR product was agarose gel-purified and subcloned into pGEM-T Easy vector (Promega) and sequenced [25].

### Preparation of synthetic miRNAs

Synthetic miRNAs were obtained from Integrated DNA Technologies. The sequence of the miRNAs were as follows: MIR-2911 5’-GGCCGGGGGACGGGCUGGGA-3’; MIR-168a 5’-UCGCUUGGUGCAGAUCGGGAC-3’; MIR168a* 5’-CCCGCCUUGCACCAAGUGAAU-3’; C7 5’-GGAUCAUCUCAAGUCUUACGU3’; C7* 5’-ACGUAAGACUUGAGAUGAUCC-3’; MIR156a 5’- UGACAGAAGAGAGUGAGCAC-3’; MIR161 5’- UCAAUGCAUUGAAAGUGACUA-3’ (asterisk denotes passenger strand). For gavage feeding, miRNAs were diluted in RNase-free PBS, and each animal was fed 400 pmols of each miRNA in 500 μL volume. For tail vein injection, the miRNAs were diluted similarly in PBS with each mouse receiving 50 fmols in 100 μL volume.

### AGO2 Immunoprecipitation

For immunoprecipitation, 250 μl of each sera sample was incubated overnight at 4°C with 3 μg of mouse monoclonal anti-AGO2 antibody (Santa Cruz Biotechnology) followed by immunoprecipitation with 25 μl of Protein L Agarose beads (Santa Cruz Biotechnology) for 4 hours at 4°C. Following purification, RNA was extracted from immunoprecipitates and unbound fractions using the miRNeasy Mini Kit (Qiagen) and 1 pmol of synthetic MIR161 was spiked into the Qiazol lysates as an exogenous RNA control. miRNA levels were quantified by qRT-PCR using TaqMan microRNA Assays as described above.

### Cultivation of fecal bacteria

Mouse fecal material was weighed and homogenized in 1X PBS to a concentration of 0.1 g/mL [26]. Fecal homogenates were then serial diluted and plated on Luria-broth plates without selection. Plates were incubated at 37°C overnight, post-incubation colony number were counted and the approximate number of bacteria per gram of feces was calculated.

### FITC-dextran permeability assay

Intestinal permeability was assessed by oral administration of FITC-dextran 4000 (Sigma). Food and water were withdrawn for 4 h, and mice were subsequently gavaged with FITC-dextran solution (60 mg/100 g body weight). Serum was collected 4 h post-gavage feeding, and FITC-dextran measurements were performed in duplicate by fluorometry (excitation, 490 nm; emission, 530nm; Cytofluor 2300, Millipore). Serial dilutions of FITC-dextran in PBS were used to calculate a standard curve.

### Drug administration

Cisplatin (Pfaltz and Bauer) was dissolved in sterile 0.9% saline at a concentration of 1 mg/mL. Mice were given a single intraperitoneal injection of either saline or cisplatin (15 mg/kg body weight).

### Statistical analysis

Statistical analyses were performed with the student T-Test formula in Microsoft Excel. Significance was set at P < 0.05. Data are presented as means ± SEMs.

## Results

### Assaying MIR2911 levels in flowers and herbs

On the basis that honeysuckle contains high levels of MIR2911, we assayed various other medicinal or edible herbs for the presence of MIR2911 by qRT-PCR (Table 1). Due to the storage issues with seasonal herbs, we restricted our survey to dried plant parts from local markets. These dried herbs and flowers were generally used as traditional Chinese medicines or as tea. Given that MIR2911 is derived from ribosomal RNA, we postulated that flowering tissues that were undergoing high levels of cell division would contain high levels of this plant-based small RNA. The sophora displayed the highest levels of MIR2911 at 6736 fmol/g while honeysuckle had 5000 fm/g. Chamomile (3056 fmol/g), blue mallow (1126 fmol/g) and ginseng flowers (277 fmol/g) displayed robust levels of MIR2911 compared to willow bark and chow (approx. 2 fmol/g). Meanwhile, hibiscus had almost no MIR2911 (0.2 fmol/g). It is notable that certain flowers, like lavender, appeared to contain substances that interfered with the RT-PCR reactions used to quantify both MIR2911 and the exogenous spike-in control. Our calculations of MIR2911 levels were based on normalization to the exogenous spike-in MIR161. If normalization was not possible, as was the case with lavender, no values were recorded.

**Table 1:** Content of MIR2911 in various herbs and flowers. Quantification of MIR2911 levels in various dried herbs and flowers. The calculation of MIR2911 concentration is based on a standard curve and normalization to the exogenous spiked-in MIR161.

Herbs & Flowers	MIR2911 (fmol/g)
Sophora	6736.2
Honeysuckle	5000
Chamomile	3056.6
Blue Mallow	1126.6
Ginseng flowers	277.8
Willow bark	2.9
Chow	2.3
Hibiscus	0.2
Lavender	ND

ND: No Detection for both MIR2911 and the spike-in MIR161 control.

### Circulating levels of plant-based MIR2911 in animals fed herbal diets

**Fig 1:**
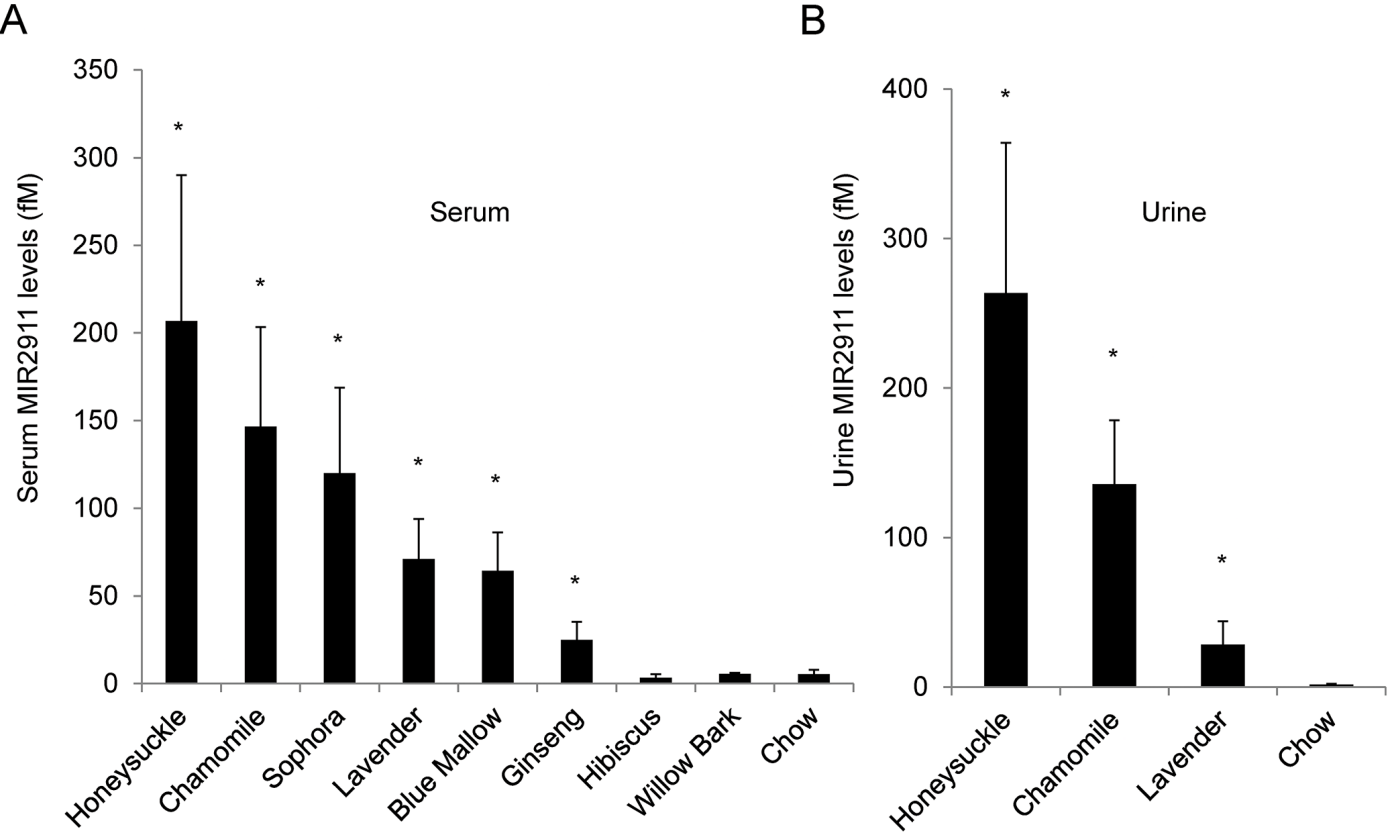
Serum and urine MIR2911 levels in mice fed various herb- or flower-containing diets. (A) Detection of MIR2911 in sera from mice fed various herb and flower diets. (B) Detection of MIR2911 in urine from mice fed various herb and flower diets. For (A) and (B), mice were fed diets for 7 days before RNA was isolated form serum and urine samples and analyzed. N = 5. Asterisk: p<0.05 between chow and herb diet fed mice. Experiment replicated at least three times with each diet.

Using a modified chow-based diet supplemented with ground herbs or flowers, we analyzed plant-derived MIR2911 levels in the sera and urine of mice seven days after initiating feeding. Fold differences in circulating MIR2911 levels in herbal diet-fed animals compared to chow-fed animals were as follows: honeysuckle, 39-fold higher (207 fM); chamomile, 27-fold higher (147 fM); sophora, 22-fold higher (120 fM); lavender, 13-fold higher (71 fM); blue mallow, 12-fold higher (64 fM); ginseng, 5-fold higher (25 fM). No significant change was detected in either hibiscus (3 fM) or willow bark (6 fM). Fold differences in the level of MIR2911 in the urine samples of mice fed herbal diets compared to chow were as follows: honeysuckle, 160-fold higher (264 fM); chamomile, 82-fold higher (135 fM); lavender, 17-fold higher (28 fM) (Fig 1). To verify the fidelity of the qRT-PCR assay kit for MIR2911 with serum samples that have relatively small quantities of MIR2911, the qPCR product was subcloned and sequenced. The presence of the full sequence of mature MIR2911 in the qPCR amplicon was confirmed.

### Testing synthetic MIR2911 serum detection

**Fig 2:**
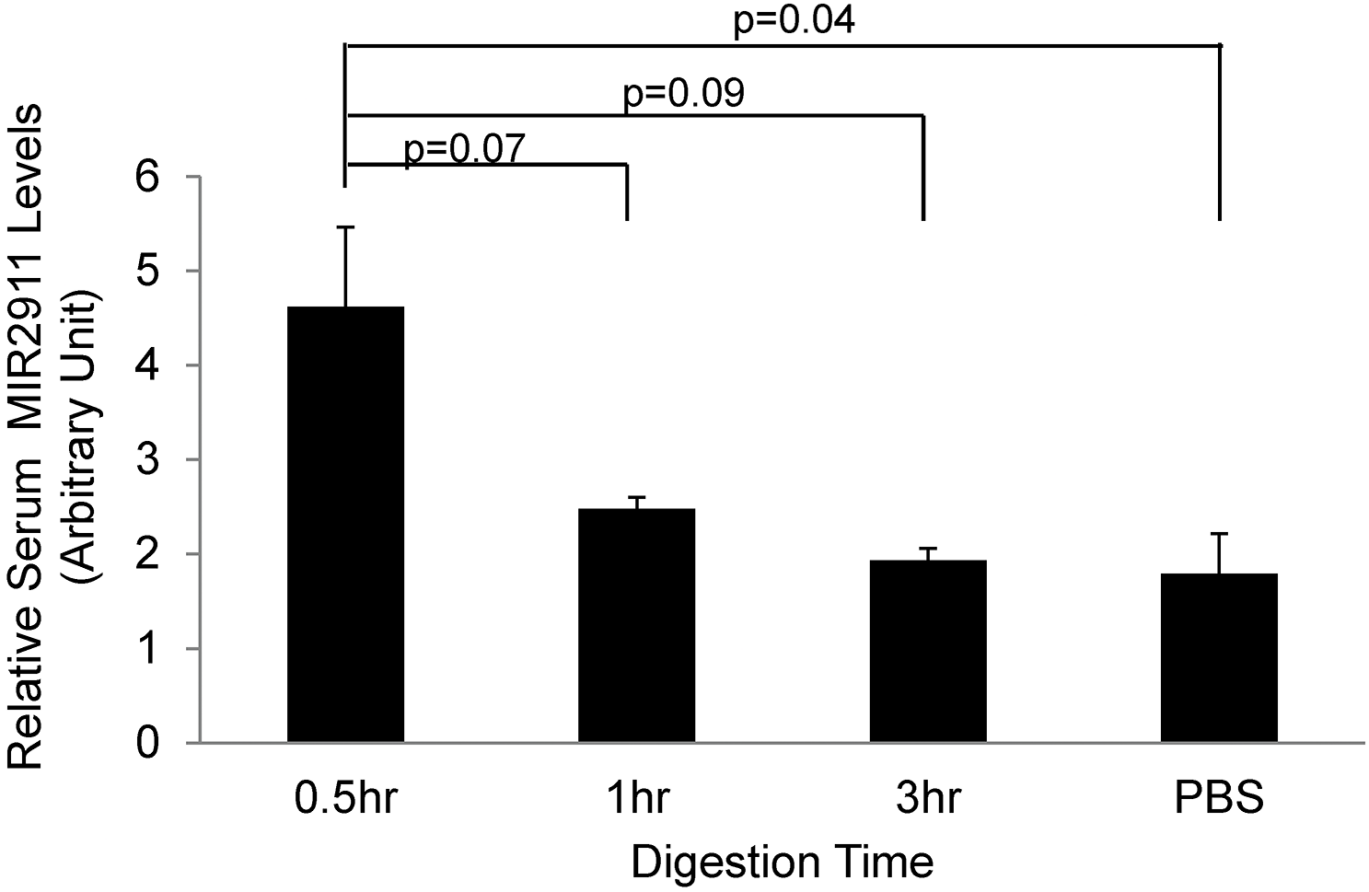
Time course analysis of absorption of gavage fed synthetic MIR2911 Time course analysis of serum MIR2911 levels in mice gavage fed 400 pmols synthetic MIR2911 at 0.5 hour, 1 hour, 3 hours after gavage feeding. Mice were pre-fed chow diet. N = 5. Experiment replicated three times.

Since we observed an approximate correlation between the dietary content of MIR2911 in the herbal and flower diets and the levels detected in sera (Table 1, Fig 2), we sought to test whether synthetic MIR2911 gavage fed at high doses can be absorbed in chow-fed animals. After single-dose feeding of 400 pmols of 2’-O methylated (plant-specific) synthetic MIR2911, serum MIR2911 levels in the mice were elevated by roughly 1-fold within 30 min after gavage feeding, but decreased to background levels approximately 1 hour after the gavage treatment (Fig 2).

### Assessing association of serum MIR2911 with AGO2

**Fig 3:**
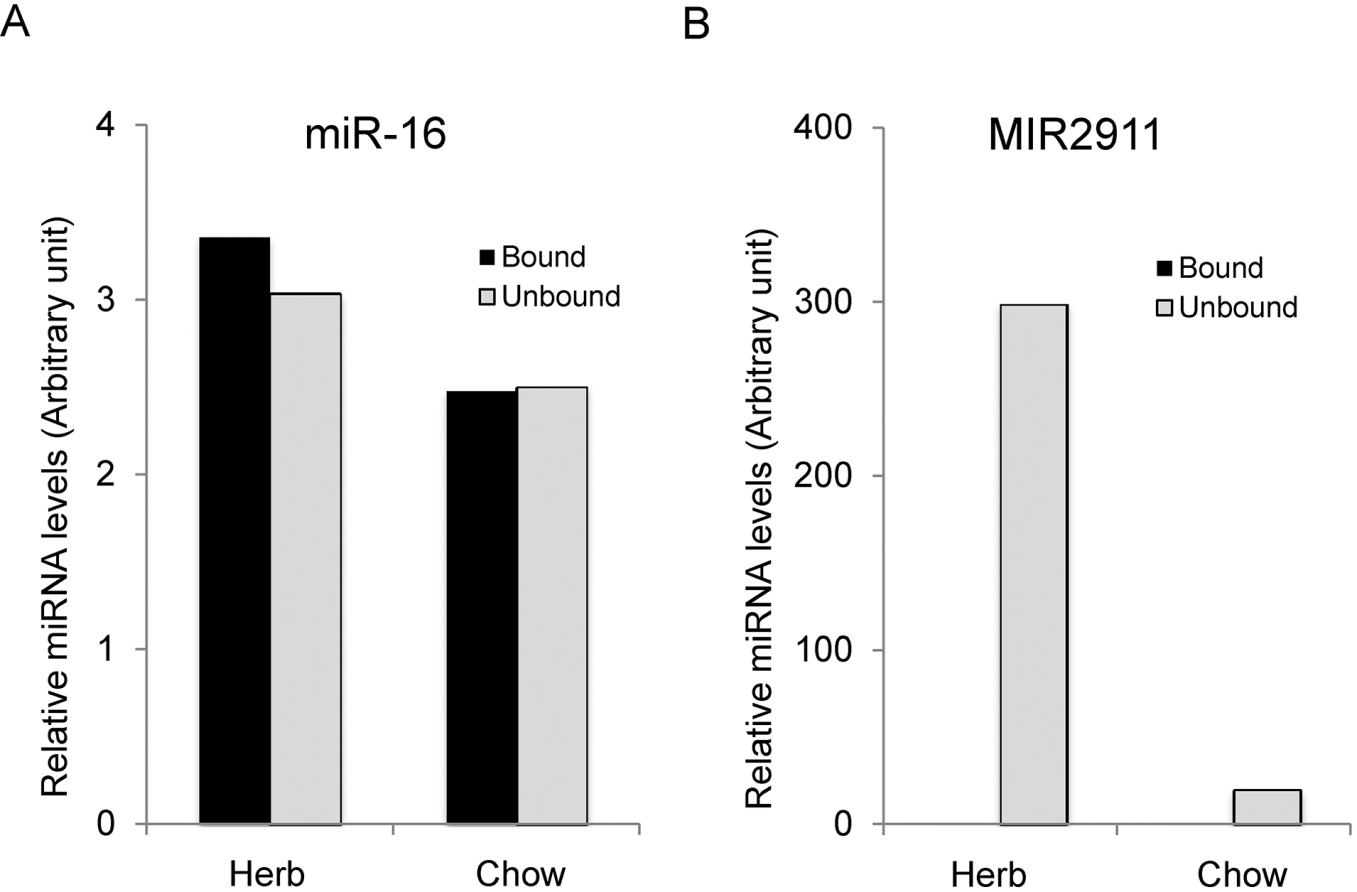
Serum MIR2911 is not associated primarily with AGO2. (A) Quantification of MIR2911 in AGO2-associated immunoprecipitates and unbound fractions of serum from animals fed an herb-based diet or a standard chow diet. (B) miR-16 immunoprecipitation was analyzed as an endogenous control for AGO2 precipitation. Levels of MIR2911 and miR-16 were each normalized to spiked-in exogenous MIR161. This experiment is representative of more than five different experiments done with herbal-fed mice (honeysuckle, chamomile).

Circulating small RNAs are resistant to RNase activity, extreme pH and temperature fluctuations [27]. One mechanism for this protection is association with an RNA-binding protein such as Argonaute 2 (AGO2) [6, 28]. We assayed the sera to determine if MIR2911 was associated AGO2 by performing co-immunoprecipitation with anti-AGO2 antibodies. Immunoprecipitation demonstrated that approximately 99% of MIR2911 was not associated with AGO2 and remained in the unbound fraction, while we recovered approximately equal concentrations of endogenous miR-16 in immunoprecipitates and the unbound fraction of sera from herb-fed animals and control mice (Fig 3).

### Clearance of circulating plant-based small RNAs

**Fig 4:**
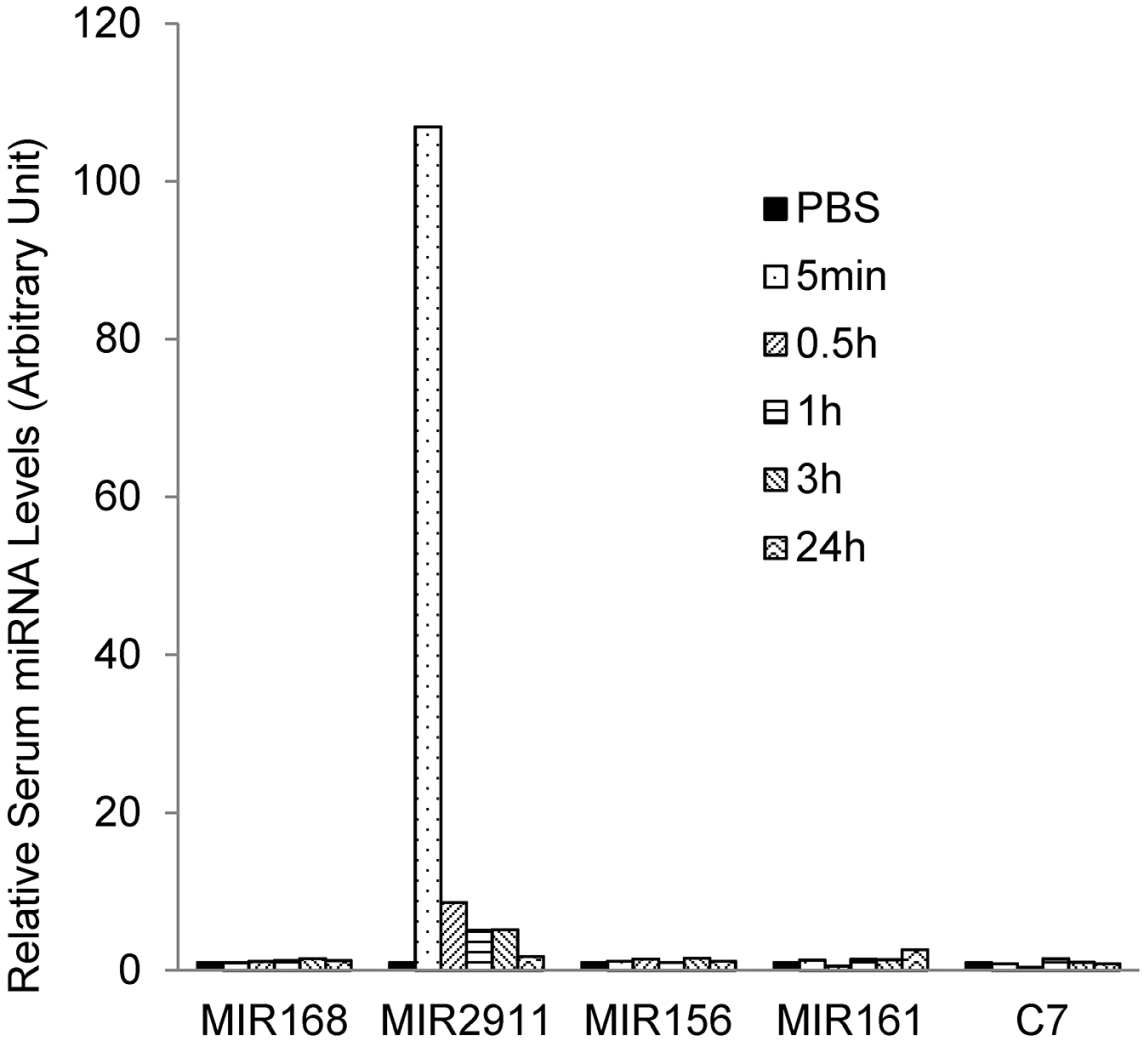
Stability of various synthetic plant microRNAs injected into mouse circulation. Time course analysis of serum microRNA levels after the injection of microRNA cocktail (5 pmol each) via lateral tail vein. 1X PBS was injected as a control. Experiment replicated more than three times.

*In vivo* delivery of small RNAs faces many challenges including limited stability in serum and rapid blood clearance [29]. Our data suggest that dietary MIR2911 is stabilized in sera without binding AGO2 (Fig 3). Could the secondary structure of MIR2911 afford protection from RNase activity in the sera? To test this notion, we assayed clearance of synthetic plant-based small RNAs directly from circulation. A cocktail of four different plant-based small RNAs (MIR2911, MIR168a, MIR156a, and MIR161) and custom designed siRNA MIRC7 were directly injected into the mouse tail vein and sera was collected from the mice at 5 min, 30 min, 1 h, 3 h, and 24 h post injection. The concentration of the intravenous dose is based on the concentrations of abundant circulatory miRNAs such as miR-16 [19]. As many previous reports have documented [29, 30], clearance of these small RNAs was rapid. Interestingly, MIR2911 levels were substantially higher at 5 minutes after injection compared to the other miRNAs administered at equal dosages. After 3 h the apparent clearance of all the small RNAs tested was complete (Fig 4).

### Impact of the gut microbiome on MIR2911 absorption

**Fig 5:**
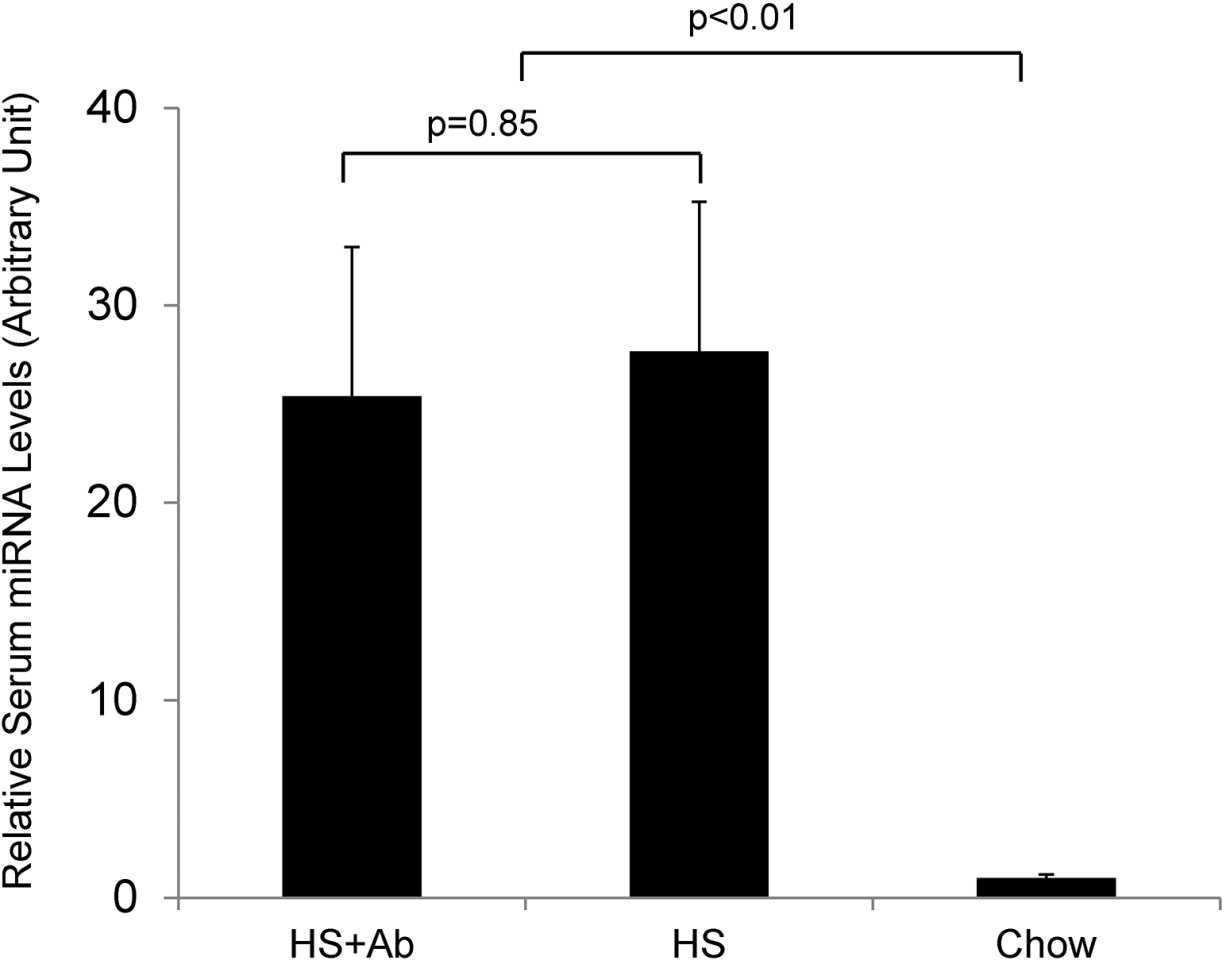
Antibiotic treatments do not appear to impact MIR2911 serum detection in honeysuckle fed mice. Analysis of serum MIR2911 levels in mice fed honeysuckle (HS), mice fed honeysuckle and treated with antibiotics (HS+Ab), and mice fed chow. Mice were fed the diets for 7 days before analysis of serum MIR2911 levels. N = 5. Experiment replicated more than three times with each diet and condition.

Gut microbes have coevolved with the host to perform a number of functions in the host animals [31]. When we examined the fecal bacterial content of animals fed honeysuckle, they exhibited more than a 100-fold increase in their microbiome titer, as measured by the increased number of colonies observed compared to chow-fed animals. This increase in bacterial titer was due to the honeysuckle and not MIR2911, as gavage feeding of the synthetic 2911 alone did not alter the microbiome content (data not shown). To test whether these microbiome changes were responsible for MIR2911 serum detection, we administered antibiotics Amoxicillin and TMS orally to mice while they were simultaneously fed the honeysuckle diet. We observed a sharp decrease in fecal bacteria titer within 1 day after beginning the antibiotic treatment. This change persisted over the course of a week. Despite the fact that the levels of fecal bacteria were suppressed to levels lower than chow-fed controls, we observed levels of MIR2911 in the sera of mice fed honeysuckle plus antibiotics that were indistinguishable from mice fed honeysuckle without antibiotics (Fig 5). We also assayed the microbiome of mice fed other herbal and flower diets. Chamomile-, sophora-, lavender-, ginseng-, and blue mallow- containing diets did not yield an increase in microbiome density compared to mice fed a chow diet (S1 Fig).

### Quantifying intestinal permeability in honeysuckle-fed animals

**Fig 6:**
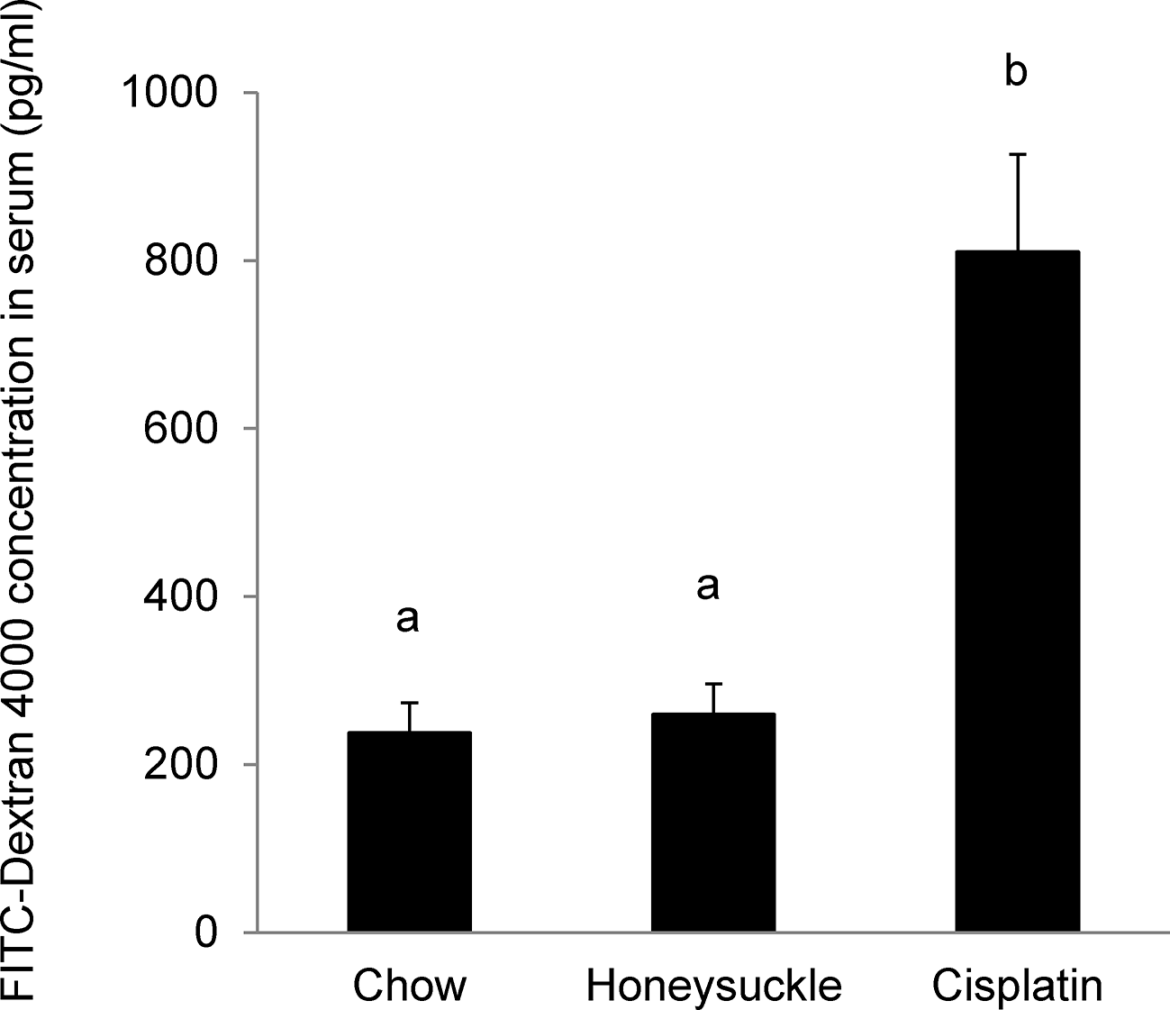
Gut permeability is not altered in honeysuckle fed mice. Quantification of FITC-Dextran 4000 translocation into the circulation of mice fed chow, honeysuckle diet, or mice fed chow diet and treated with cisplatin. N = 5. p (a/b) < 0.01. Experiment replicated three times.

To examine potential intestinal changes caused by honeysuckle feeding that may potentiate small RNA uptake, we used the non-metabolizable macromolecule FITC-dextran 4000 as a permeability probe [32]. Integrity of the gut epithelial barrier in honeysuckle-fed animals was assessed by gavage feeding mice with the 4 kD FITC-dextran molecule and measuring its translocation into circulation. We detected no difference in permeability to FITC-dextran in the honeysuckle-fed animals compared to the chow-fed animals. Cisplatin treatment was used as a positive control for increased permeability, as this chemical is known to promote increased gut permeability [33] (Fig 6).

## Discussion

By analyzing animals fed numerous plant-based diets high in MIR2911, we demonstrated consistent dietary delivery of a plant-based small RNAs into circulation. Previously, we demonstrated the dietary origins of this small RNA using digital drop PCR [19], and here we have sequenced the qPCR product to further validate that this is an exogenous RNA. Given the controversy surrounding this area of inquiry [1], we have focused our efforts here on dietary components and uptake parameters. We realize there are differences in the nutritional content of our herbal diets (Table 1); however, we consistently observed an association between plant MIR2911 content and circulatory MIR2911 levels in the consumer (Table 1, Fig 1). Furthermore, animals fed diets low in MIR2911 (willow bark, hibiscus, chow) never displayed circulating levels of the diet-based small RNA above background levels.

The serum MIR2911 levels after honeysuckle feeding (max. levels 207 fM) reported here are almost 6-fold lower than those recently reported by a group using gavage feeding [18]. Our results demonstrated that in sera the majority of the circulating MIR2911 was not bound by AGO2 (Fig 3). Conversely, the honeysuckle gavage feeding study suggests that the majority of MIR2911 is associated with AGO2 [18]. Future work will have to discern if these differences are due to the dietary delivery or procedural differences between the labs.

We have documented increased circulating levels of MIR2911 when using diets containing honeysuckle, sophora, chamomile, lavender, blue mallow and ginseng. The amount of small RNAs in the food is certainly an important component when considering the efficacy of detection. It has been shown that cow’s milk contains an assortment of abundant miRNAs, and nutritionally relevant amounts of cow’s milk may be sufficient to alter human gene expression [15]. Like our MIR2911-containing plant foods, the milk dosages of miRNAs are many times higher than those of the rice miRNAs reported to alter liver function [10]. No locus in the mouse genome appears to encode for a MIR2911-like small RNA [19], thus unlike the milk studies, our study is not complicated by ambiguities regarding assignment of small RNA origin between the dietary sources and the consumer [34]. This milk study also discounted the relevance of plant-based miRNAs when they failed to detect circulating levels of a single plant-derived miRNA in consumers. It should be noted that the vegetables they fed to the consumers, for a single serving, contained only 50 fmol/g of the measured miRNA, about 1/100 the concentration of MIR2911 in honeysuckle (Table 1). A new paradigm of cell-to-cell transfer of circulating miRNAs has been proposed and demonstrated in immune cell types [35]. This studies demonstrates that femtomolar amounts of a specific miRNA alters the fate of a targeted cell [35]. This work may change the way we view circulating miRNAs, as it demonstrates that currently undetectable amounts of miRNAs can have biological functions.

Previous work has suggested that MIR2911 is remarkably stable during RNase treatment and boiling [18]. In agreement with these observations, we found that MIR2911 was more stable than the other plant-based miRNAs tested in circulation following administration via tail vein injection (Fig 4). The stability of MIR2911 may be due to its high GC content. In fact, experiments that lower the GC content appear to decrease the half-life of this RNA [18]. The uptake and functionality of dietary RNAs in consuming populations faces major obstacles including stability of the RNA and delivery across cellular barriers [36]. By comparing the stability of ingested plant-based small RNAs versus those directly injected into the circulation, we can infer that the processes of absorption and transportation protect dietary small RNAs. This scenario has been proposed by others [18] and certainly warrants further mechanistic studies.

Our previous study proposes a model where gut and kidney perturbations facilitate detection of plant-based miRNAs in sera of consumers [19]. The gut microbiome can rapidly switch between herbivorous and carnivorous functional profiles [37], and we speculated that certain bacteria might facilitate the uptake of plant-based small RNAs [38]. However, while the honeysuckle diet altered fecal bacteria counts, this change did not appear to impact dietary transfer of MIR2911 (Fig 5). In agreement with this finding, other herbal diets such as chamomile and sophora did not appear to alter the animal microbiome but still facilitated the serum detection of MIR2911 (Fig 1 and S1 Fig). However, our studies do not rule out the possibility that gut bacteria are required for dietary small RNA uptake. In fact, extracellular bacterial RNAs may also participate in intercellular communication [38]. In the future, more meaningful insights may be obtained using molecular tools, e.g. high-throughput 16S rRNA amplicon sequencing and/or anaerobic culturing, and more clearly defined antibiotic regimes.

The intestine allows the absorption of nutrients while simultaneously functioning as a barrier [39]. We previously demonstrated that gut architecture is not modified by honeysuckle feeding [19], consequently we tested whether the herb-based diets could alter the permeability of the gut to facilitate serum detection, and we demonstrated that permeability is not altered in the honeysuckle fed animals (Fig 6). In fact, our data suggest that a single large oral dose (400 pmol) of synthetic MIR2911 into a healthy animal is sufficient to promote short-term serum detection (< 30 min), though only at a small fold difference compared to the non-gavage fed controls (Fig 2). This gavage dosage of MIR2911 is equivalent to 40 days of mice eating the honeysuckle diet, thus we venture the difference in serum detection between the diets and gavage fed animals could be due to metabolic changes that affect uptake of dietary RNAs occurring during the prolonged feeding. Alternatively, the MIR2911 from plant materials maybe better protected from digestion than synthetic forms in the GI tract, or persistent presentation of MIR2911 via food particles in the GI tract ensures a constant input level at the intestinal epithelial interface, thus facilitating more efficient absorption. Certainly given that MIR2911 is not associated with AGO2 (Fig 3), this study leaves open the possibility that MIR2911 is protected after uptake within exosomes. Could MIR2911 be protected by plant derived exosome-like nanoparticles [40] or are these exosomes derived from the consumer? Regardless, our findings here suggest that under specific dietary conditions, no gut pathology is required to facilitate uptake.

Dietary small RNAs may be differentially absorbed and circulated within the consumer. Certainly the functionality of both endogenous and dietary circulating small RNAs remains controversial [41]. Nonetheless, we should not disparage the dietary-RNA concept based solely on the failure to replicate initial findings [10]. A valuable lesson can be drawn from the work presented here: high dosages of food-based RNAs facilitated transmission to healthy consumers; we speculate that dietary uptake of RNAs will be effective through prolonged exposure to elevated levels of highly stable small RNAs. Considering the impact that dietary small RNAs could have on nutrition, health-care [18, 42], and agbiotechnology further work regarding the nuances of dietary RNA delivery and functionality are warranted.

## Supporting Information

S1 Fig Fecal bacteria titer from mice fed various herbal and flower diets. Fecal bacterial titer for mice on various herbal and flower diets were measured after mice were fed 7 days.
(TIF)
