# Supplementary figures and images for: Detection of an Abundant Plant-Based Small RNA in Healthy Consumers
Source: PLoS One. 2015 Sep 3;10(9):e0137516. doi: 10.1371/journal.pone.0137516 (PMC4559308; doi:10.1371/journal.pone.0137516)

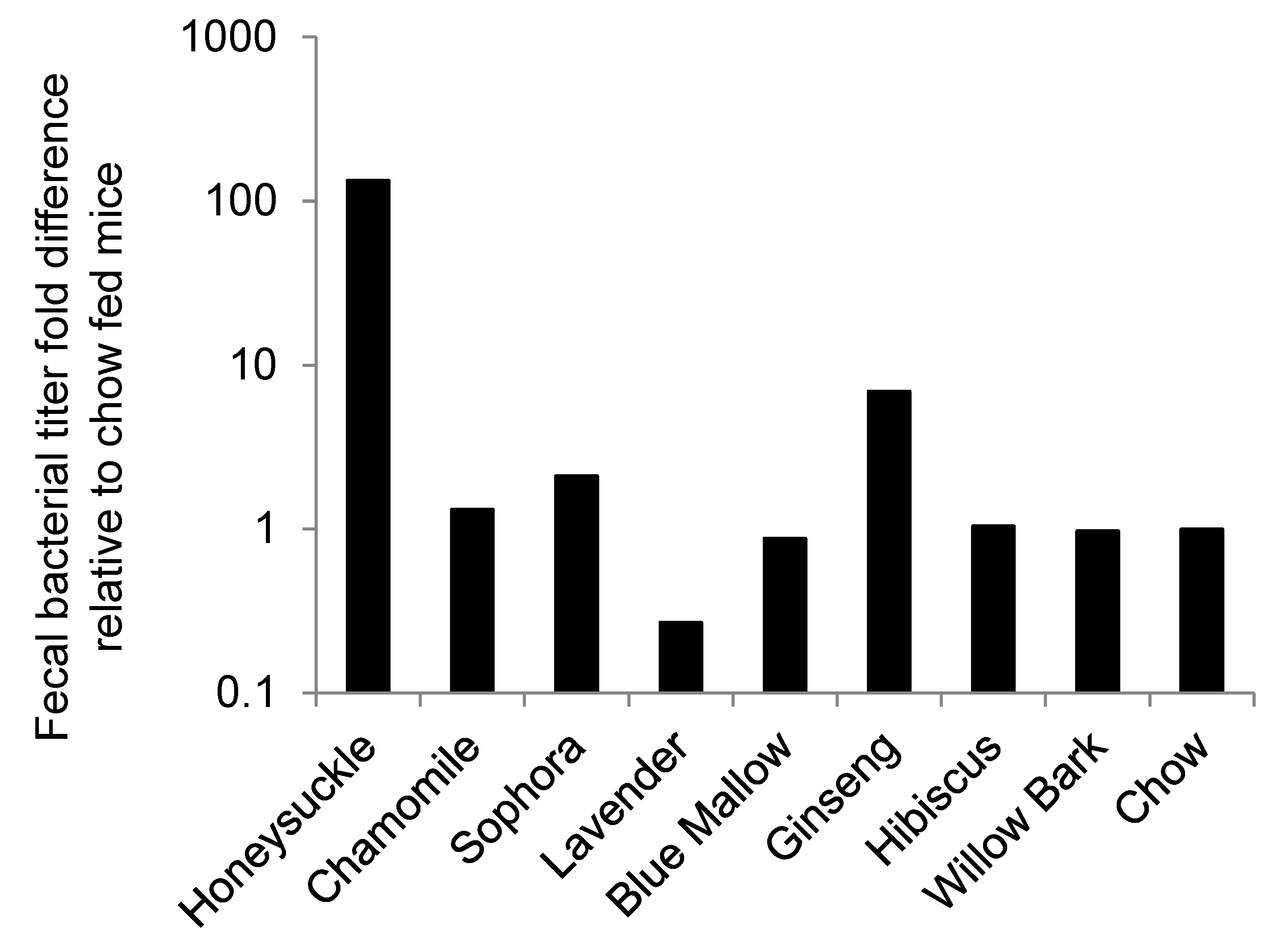

Supplement: S1 Fig — Fecal bacterial titer for mice on various herbal and flower diets were measured after mice were fed 7 days. (TIF) [file pone.0137516.s001.tif]
